# p53 loss-of-heterozygosity is a necessary prerequisite for mutant p53 stabilization and gain-of-function *in vivo*

**DOI:** 10.1038/cddis.2017.80

**Published:** 2017-03-09

**Authors:** Evguenia M Alexandrova, Safia A Mirza, Sulan Xu, Ramona Schulz-Heddergott, Natalia D Marchenko, Ute M Moll

**Affiliations:** 1 Department of Pathology, Stony Brook University, Stony Brook, NY, USA; 2 Institute of Molecular Oncology, University of Göttingen, Göttingen, Germany

## Abstract

Missense mutations in *TP53* comprise >75% of all p53 alterations in cancer, resulting in highly stabilized mutant p53 proteins that not only lose their tumor-suppressor activity, but often acquire oncogenic gain-of-functions (GOFs). GOF manifests itself in accelerated tumor onset, increased metastasis, increased drug resistance and shortened survival in patients and mice. A known prerequisite for GOF is mutant p53 protein stabilization, which itself is linked to aberrant protein conformation. However, additional determinants for mutant p53 stabilization likely exist. Here we show that in initially heterozygous mouse tumors carrying the hotspot GOF allele R248Q (p53Q/+), another necessary prerequisite for mutant p53 stabilization and GOF *in vivo* is loss of the remaining wild-type p53 allele, termed loss-of-heterozygosity (LOH). Thus, in mouse tumors with high frequency of p53 LOH (osteosarcomas and fibrosarcomas), we find that mutant p53 protein is stabilized (16/17 cases, 94%) and tumor onset is significantly accelerated compared with p53+/− tumors (GOF). In contrast, in mouse tumors with low frequency of p53 LOH (MMTV-Neu breast carcinomas), mutant p53 protein is not stabilized (16/20 cases, 80%) and GOF is not observed. Of note, human genomic databases (TCGA, METABRIC etc.) show a high degree of p53 LOH in all examined tumor types that carry missense p53 mutations, including sarcomas and breast carcinomas (with and without HER2 amplification). These data – while cautioning that not all genetic mouse models faithfully represent the human situation – demonstrate for the first time that p53 LOH is a critical prerequisite for missense mutant p53 stabilization and GOF *in vivo*.

Missense mutations in *TP53* (mutp53) comprise >75% of all p53 alterations in cancer, resulting in highly stabilized mutant p53 proteins that not only lose their tumor-suppressor activity, but often acquire oncogenic gain-of-functions (GOFs).^1, 2, 3, 4, 5^ GOF activities promote cancer metabolism, stemness, and malignant progression and invasion. This results in accelerated tumor onset, increased metastasis, increased drug resistance and shortened survival in patients and mice.^5, 6, 7^ Accordingly, mutp53 knockin mice carrying the human hotspot missense R248Q mutation have significantly earlier tumor onset and shorter survival than p53-null mice.^5^ In agreement, in human patients with sporadic cancers across six major tumor entities, cancers with GOF mutp53 R282 and R248 alleles show a twofold higher hazard ratio (i.e., increased mortality) compared with cancers with loss-of-function (LOF) mutp53 alleles (nonsense, frameshift and deletion mutations).^8^ Similarly, germline Li–Fraumeni syndrome (LFS) patients carrying R248Q mutp53 exhibit markedly faster tumor onset by 10.5 years and higher tumor numbers per person than LFS patients carrying LOF mutp53.^5^

Conversely, mutp53 elimination significantly suppresses tumor growth and metastasis and markedly extends survival in various mouse models.^7, 9, 10^ For example, mutp53 depletion by RNAi has strong cytotoxic effects in human cancer cell lines *in vitro* and in xenografts.^7^ In allografts, knockdown of mutp53 in K*ras*^G12D^ pancreatic cancer cells strongly reduces their metastatic ability.^9^ Finally, in a conditional inactivatable (‘floxable') autochthonous mouse model, ablation of the R248Q knockin allele extends survival by 37%, induces regression or stagnation of advanced tumors and strongly suppresses metastasis.^10^

A known prerequisite for mutp53 GOF is its massive constitutive protein stabilization specifically in tumors – but not in normal cells – of knockin mice.^6, 11, 12^ Currently about 11 million patients worldwide live with cancers expressing highly stabilized mutp53, raising the question: what factors determine mutp53 stabilization leading to oncogenic GOF? One established determinant are the aberrant protein conformations of both the structural and DNA-contact classes of missense mutant p53 proteins, requiring constitutive chaperone complexing (with, e.g., Hsp90 and Hsp40) to protect them from their E3 ubiquitin ligases Mdm2 and CHIP and proteasomal degradation.^10, 13, 14, 15, 16, 17, 18^ Indeed, pharmacological inhibition of the HSP90 chaperone machinery destabilizes mutp53, leading to 48% and 59% prolonged survival in R175H and R248Q knockin mice, respectively.^10^ We hypothesized that besides aberrant conformation additional determinants of mutp53 stabilization likely exist. Here we show that loss of the remaining wild-type p53 (wtp53) allele, termed loss-of-heterozygosity (LOH), is also critical for missense mutp53 stabilization and GOF *in vivo*.

## Results

**Figure 1:**
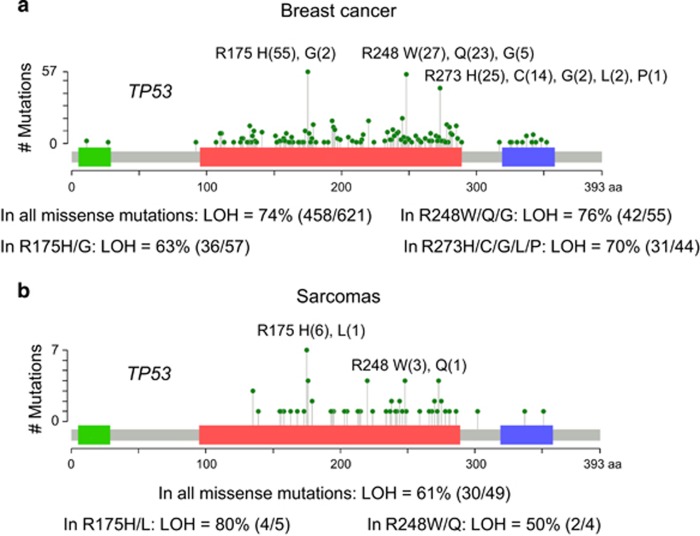
Analysis of the databases of sporadic human breast cancer (**a**) and sarcomas (**b**) (METABRIC, provisional TCGA and others, see Materials and Methods section) show a high frequency of wtp53 allele loss (LOH) in missense mutp53 tumors

TCGA, METABRIC and other databases of sporadic human cancer show wtp53 allele loss (LOH) in the majority of missense mutp53 tumors, including ovarian cancer, breast cancer and sarcomas (Figure 1, Tables 1 and 2). Specifically, in human HER2 breast cancer with concomitant missense mutp53, wtp53 LOH occurs in 82.3% of patients (Table 1). Thus, we hypothesized that LOH is a second determinant of mutp53 stabilization and GOF *in vivo*.

**Figure 2:**
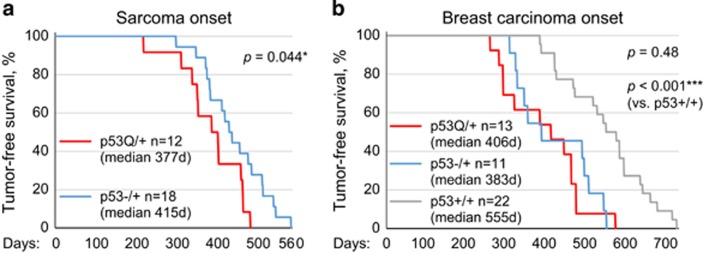
Survival curves analyzing tumor onset of sarcomas and breast carcinomas in p53Q/+Neu, p53−/+Neu and p53+/+Neu mouse cohorts. (**a**) Sarcoma onset is faster in p53Q/+Neu compared with p53−/+Neu mice. This indicates either a DN effect over wtp53 or, alternatively, p53 LOH resulting in mutp53 GOF specifically in sarcoma. (**b**) Breast cancer latency in p53Q/+Neu and p53−/+Neu siblings is similar, reflecting that the majority of p53Q/+ breast tumors did not undergo LOH (see Figure 3b) in contrast to human breast cancer, and also did not exert a DN effect over wtp53 but simply behaved as a LOF allele. Kaplan–Meier analysis;*n*, number of mice; *P*, log rank statistics

To test this, we combined the heterozygous hotspot GOF allele R248Q (‘p53Q/+')^5, 10^ with the MMTV-Neu (‘Neu') oncogene^19^ expressing additional wild-type HER2 copies selectively in the mammary gland, as mutp53 has a strong prognostic value in HER2-positive breast cancer, that is, significantly increased mortality.^20^ Although p53Q/+Neu mice developed breast cancer faster than p53+/+Neu mice, surprisingly breast cancer latency between p53Q/+Neu and p53−/+Neu siblings was similar (Figure 2b), suggesting that mutp53 R248Q did not exert a dominant-negative (DN) effect over wtp53 but simply behaved as a LOF allele in Neu-driven breast tumorigenesis *in vivo*, hence the curves overlap.

**Figure 3:**
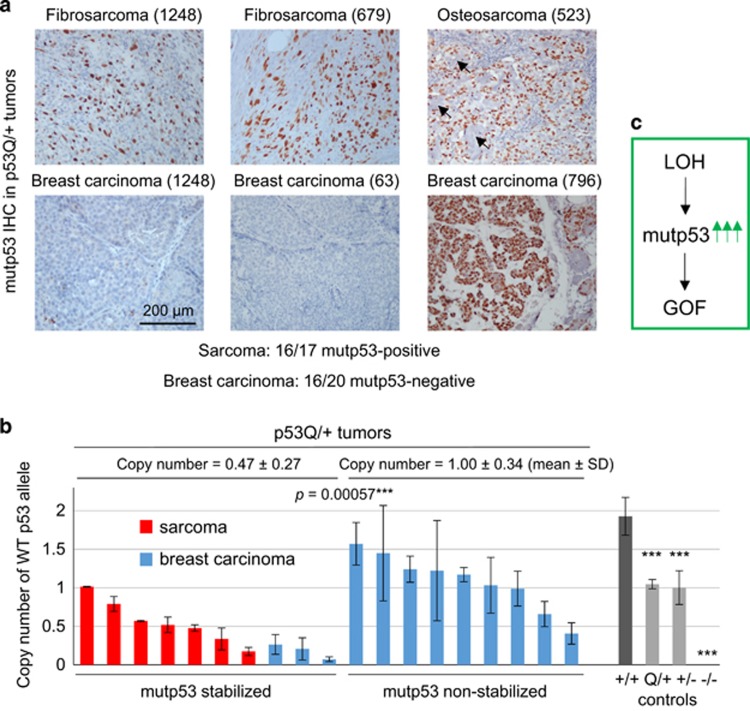
Loss of wtp53 allele is required for missense mutant p53 stabilization and GOF. (**a**) The vast majority of sarcomas (16/17 cases, 94%) have stabilized mutp53. In contrast, the majority of breast carcinomas (16/20 cases, 80%) do not. Immunohistochemistry for mutp53. Mouse identity in parentheses. Arrows indicate the osteoid in osteosarcoma. (**b**) Analysis of wtp53 copy number in sarcomas and breast carcinomas of p53Q/+Neu mice by quantitative genotyping. Tumors with mutp53 stabilization (all sarcomas and three breast cancers tested) have significantly higher LOH than tumors without mutp53 stabilization (majority of breast cancers). Note, as sarcomas have high normal stroma contamination (top, blue mutp53-negative stromal cells, which do not have LOH), the actual LOH in sarcomas is most likely even higher because of dilution of the tumor genotype, causing LOH underestimation. For the same reason, copy numbers of the two highest sarcoma cases (two left red bars) are likely inflated. The wtp53 signal was normalized to the Rosa26 signal. Tail biopsies from p53+/+ (two wt alleles), p53Q/+, p53−/+ (one wt allele) and p53−/− mice (no wt alleles) were used as normal control tissues without LOH. Bars represent mean±S.D. of two technical replicas of individual cases. ***P<0.001. (**c**) Schematic diagram of the proposed mechanism for mutp53 stabilization and GOF in heterozygous tumors. Loss of the wtp53 allele (LOH) causes accumulation of highly stabilized mutp53 protein, which triggers tumor development and is the principle mechanism and prerequisite of GOF

However, about half of p53Q/+Neu and p53−/+Neu mice did not develop breast cancer but instead developed osteosarcomas and fibrosarcomas, which originate from mesenchymal tissues where MMTV-Neu is not expressed (Figure 2a). Notably, sarcoma onset was faster in p53Q/+Neu compared with p53−/+Neu mice, indicating either a DN effect of mutp53 over wtp53 or, alternatively, wtp53 LOH resulting in mutp53 GOF specifically in sarcoma. Importantly, this survival difference between sarcoma and breast cancer correlated with mutp53 stabilization in nearly all examined sarcomas (94%, 16/17), but only in rare breast carcinomas (20%, 4/20), even within the same animal (Figure 3a, e.g., animal #1248).

**Figure 4:**
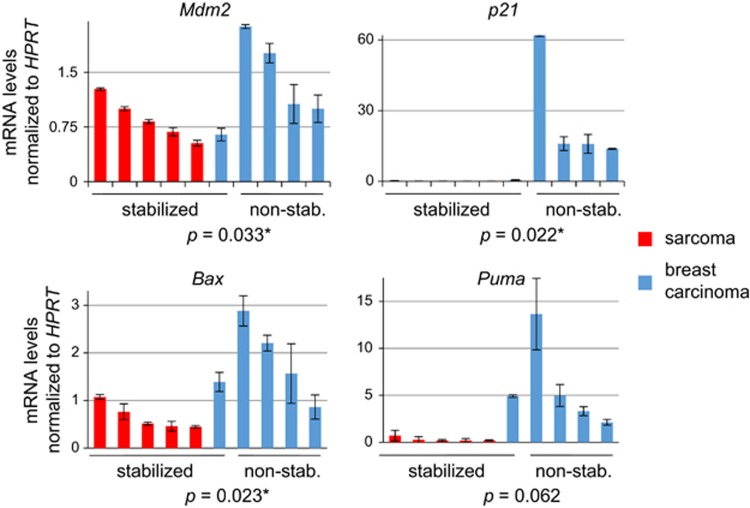
Real-time qPCR analysis of wtp53 target genes *Mdm2, p21, Bax* and *Puma* shows that their expression is largely decreased in samples with mutp53 stabilization compared with samples without mutp53 stabilization

Thus, we asked whether sarcomas are more prone to p53 LOH than breast tumors. Indeed, qPCR of genomic DNA showed that p53 LOH occurs in all sarcomas, but rarely in breast cancer (Figure 3b). Moreover, the few breast tumors that did stabilize mutp53 also underwent p53 LOH. Together, this strongly suggests that LOH is a critical prerequisite for mutp53 stabilization and GOF (Figure 3c). To corroborate our LOH data, we analyzed p53 target genes as another readout for the remaining wtp53 allele activity (Figure 4). Indeed, all tumors with stabilized mutp53, including the single ‘outlier' breast cancer tested, had reduced or undetectable *Mdm2* and *p21* levels, respectively, and sarcomas also had reduced *Bax* and *Puma* expression correlating with their LOH.

## Discussion

In sum, we propose that p53 LOH is a necessary prerequisite for mutp53 stabilization and GOF activity *in vivo* (Figure 3c). Indeed, we find that *TP53* LOH is a frequent event in human cancers with missense mutp53, including sarcomas (61%), breast cancer with or without HER2 amplification (up to 82%) and ovarian cancer (75%) (Figure 1, Tables 1 and 2). This high LOH frequency coincides with mutp53 protein stabilization^21, 22^ and GOF in human cancers.^5, 8^ Our *TP53* LOH data are in agreement with earlier reports finding 60% *TP53* LOH in LFS patients,^23^ 81% in sporadic breast cancer patients (all molecular subtypes pooled)^20^ and 93% across 10 sporadic human cancer types,^24^ all expressing missense mutp53. Note that the latter study with the highest frequency includes ‘copy neutral' *TP53* LOH (defined as reduced wtp53 mRNA expression but genomic copy present) and also corrects for dilutional effects from stromal contamination.^24^ This suggests that conventional and even quantitative real-time PCR – which we used in our analysis – likely underestimate true functional p53 LOH.

In full agreement with the human data, sarcomas in our mouse model also exhibit GOF because they undergo LOH, which enables mutp53 stabilization. Similarly, Shetzer *et al.*^25^ found that isolated mesenchymal stem cells from heterozygous R175H/+ mice form subcutaneous tumors only after they undergo wtp53 LOH. How mechanistically p53 LOH induces mutp53 stabilization awaits further investigation. A possible contributor could be reduced expression of the wtp53 target gene *Mdm2* (Figure 4), the main ubiquitin ligase for both wtp53 and mutp53.^11, 16^

Although a few murine breast cancer cases in our MMTV-Neu model (4/20) did undergo LOH and exhibited mutp53 stabilization, for unknown reasons the majority (16/20) lacked LOH and therefore lacked mutp53 stabilization. We speculate that the pressure for p53 LOH is eliminated because of the Neu oncogene. This gives us pause that not all mouse models faithfully mimic the human genetic constellation for every tissue type, as the MMTV-Neu model contrasts with human breast cancer, which exhibit prominent LOH despite the presence of other oncogenic drivers (Figure 1a, Table 1).^20^

## Materials and methods

### *TP53* LOH analysis in sporadic human cancers

*TP53* LOH in sporadic human cancers was analyzed using the cBioPortal tool (www.cbioportal.org). The breast cancer data set included METABRIC and provisional TCGA databases, with 3014 samples with known mutant p53 status in total. The sarcoma data set included provisional TCGA, MSKCC/Broad Institute, Institut Curie and other databases, with 710 samples with known mutp53 status in total.

### Animals

Hotspot knockin mice harboring human exons 4–9 and the p53 R248Q missense mutation (‘Q' allele) and p53−/− mice, both on pure C57Bl6 background, were previously described.^5, 10^ MMTV-Neu (‘Neu') transgenic mice on pure FVBN background were from Jackson Laboratories (Bar Harbor, ME, USA) (FVB/N-Tg(MMTVneu)202Mul/J).^19^ To obtain p53Q/+Neu/+ and control p53+/−Neu/+ mice, parental p53 R248Q/+ and p53−/+ strains were first crossed to obtain p53 R248Q/- mice, followed by cross with Neu/Neu mice. Only female mice were used for all experiments. Animals were monitored weekly to determine their breast cancer and sarcoma onset and were promptly killed when their tumors reached 2 cm^3^ in volume or when animals appeared moribund. All animals were treated humanely and according to the guidelines issued by the Institutional Animal Care and Use Committee (IACUC) at Stony Brook University.

### Immunohistochemistry and histology

For immunohistochemical analysis, freshly dissected tissues were formalin fixed, paraffin embedded and sectioned (5 *μ*m). Slides were deparaffinized and boiled in citrate buffer (10 mM, pH 6.0, 35 min) for antigen retrieval, blocked in 10% goat serum and incubated with the primary antibody (mutp53, Santa Cruz, Dallas, TX, USA, FL393, sc-6243, dilution 1:500) for 2 h at room temperature. After PBS washing, slides were incubated with biotinylated secondary antibody and HRP-streptavidin using the Histostain SP Broad Spectrum kit (Invitrogen, Carlsbad, CA, USA, 959943B), stained with DAB substrate with hematoxylin counterstain and coverslipped. In addition, cancer type (breast cancer *versus* osteosarcoma or fibrosarcoma) was determined by H&E staining (data not shown).

### Quantitative LOH analysis

Genomic DNA was isolated from sarcomas, breast carcinomas and control tails using DNeasy Blood and Tissue kit (Qiagen, 69506) and quantified by spectrophotometer. Quantitative real-time PCR was performed in duplicates with QuantiTect SYBR Green Mix (Qiagen, Germantown, MD, USA, 204143) on the MJ Research DNA Engine Opticon 2 machine, using 8 ng genomic DNA and the following mouse wtp53 allele-specific primer pairs: 5′-ACAGCGTGGTGGTACCTTAT-3′ (forward) and 5′-TATACTCAGAGCCGGCCT-3′ (reverse). These wtp53 primers anneal to mouse exons 5 and 6 and do not recognize the humanized mutp53 allele. For all samples, the wtp53 signal was normalized to the Rosa26 signal measured by the following primers: 5′-AAAGTCGCTCTGAGTTGTTAT-3′ (forward) and 5′-GGAGCGGGAGAAATGGATATG-3′ (reverse).

### Statistical analysis

Kaplan–Meier analysis and log rank statistics was used to analyze tumor onset. Unpaired two-tailed Student's *t*-test was used to analyze p53 LOH and expression of p53 target genes. *P<0.05, ***P<0.001.

## Figures and Tables

**Table 1:** Frequency of p53 LOH in human HER2-positive breast cancer carrying concomitant missense mutp53

**Database**	**Cases with p53 LOH**	**Total number of cases**	**LOH frequency**
METABRIC	97	124	78.2%
TCGA provisional	38	40	95.0%
Total	135	164	82.3%

**Table 2:** Frequency of p53 LOH in human high-grade serous ovarian adenocarcinoma carrying concomitant missense mutp53

**Database**	**Cases with p53 LOH**	**Total number of cases**	**LOH frequency**
TCGA provisional	206	274	75.2%
